# A comparison of small monetary incentives to convert survey non-respondents: a randomized control trial

**DOI:** 10.1186/1471-2288-11-81

**Published:** 2011-05-26

**Authors:** Joan M Griffin, Alisha Baines Simon, Erin Hulbert, John Stevenson, Joseph P Grill, Siamak Noorbaloochi, Melissa R Partin

**Affiliations:** 1 Center for Chronic Disease Outcomes Research (CCDOR), Minneapolis VA Health System, One Veterans Drive, Minneapolis, MN 55417, USA; 2 Department of Medicine, University of Minnesota Medical School, 420 Delaware Street SE, MMC 194, Suite 14-110 Phillips-Wangensteen Bldg, Minneapolis, Minnesota 55455, USA; 3 University of Wisconsin Survey Center, University of Wisconsin, Sterling Hall, 475 N. Charter St. Madison, WI 53706-1582, USA

**Keywords:** Cost-effectiveness, data collection, incremental cost-effectiveness ratios, financial incentives, response rate, non-response

## Abstract

**Background:**

Maximizing response rates is critically important in order to provide the most generalizable and unbiased research results. High response rates reduce the chance of respondents being systematically different from non-respondents, and thus, reduce the risk of results not truly reflecting the study population. Monetary incentives are often used to improve response rates, but little is known about whether larger incentives improve response rates in those who previously have been unenthusiastic about participating in research. In this study we compared the response rates and cost-effectiveness of a $5 versus $2 monetary incentive accompanying a short survey mailed to patients who did not respond or refused to participate in research study with a face-to-face survey.

**Methods:**

1,328 non-responders were randomly assigned to receive $5 or $2 and a short, 10-question survey by mail. Reminder postcards were sent to everyone; those not returning the survey were sent a second survey without incentive. Overall response rates, response rates by incentive condition, and odds of responding to the larger incentive were calculated. Total costs (materials, postage, and labor) and incremental cost-effectiveness ratios were also calculated and compared by incentive condition.

**Results:**

After the first mailing, the response rate within the $5 group was significantly higher (57.8% vs. 47.7%, p < .001); after the second mailing, the difference narrowed by 80%, resulting in a non-significant difference in cumulative rates between the $5 and $2 groups (67.3% vs. 65.4%, respectively, p = .47). Regardless of incentive or number of contacts, respondents were significantly more likely to be male, white, married, and 50-75 years old. Total costs were higher with the larger versus smaller incentive ($13.77 versus $9.95 per completed survey).

**Conclusions:**

A $5 incentive provides a significantly higher response rate than a $2 incentive if only one survey mailing is used but not if two survey mailings are used.

## Background

In survey research maximizing response rates is critically important in order to provide the most generalizable and unbiased results. High response rates reduce the chance of respondents being systematically different from non-respondents, and thus, reduce the risk of results not truly reflecting the study population. Even small proportions of non-response have been shown to bias study findings and lead to spurious conclusions[1]. Offering nominal financial incentives for participating in survey research is a common practice and often a cost-effective method to improve response rates[2-4]. While some, including institutional review boards, have questioned whether monetary incentives provide an inappropriate influence on potential participant's decisions to participate or not[5,6], evidence suggests that nominal incentives are a harmless approach for improving response rates[7]. Systematic reviews of randomized trials of monetary incentives have found that response rates increase when: 1) any incentive is offered versus no incentive[2]; 2) incentives are unconditional (incentives are pre-paid with survey mailing and not dependent on survey completion)[2]; and, 3) larger versus smaller monetary incentives are sent[8]. Studies have also shown that incentives versus no incentives increase response rates for certain populations that typically have high rates of refusals in research studies, and therefore are underrepresented in research, including those with lower levels of education, income[9,10] and non-whites[9,11]. Less is known, however, about whether larger incentives improve response rates among people who have refused requests to participate in previous research[12-15] or whether incentives in such groups are cost-effective [10,16-19].

In this study we examine how different cash incentives ($5 versus $2) attached to an ancillary mailed survey affect response rates in a group of patients who either passively or actively declined participation in a face-to-face survey with a $25 incentive. The primary study hypothesis was that those randomly chosen to receive a $5 incentive would be more likely to respond than those who received a $2 incentive and that the $5 incentive would be more cost effective than the $2 incentive. We also hypothesized that, compared to $2, a $5 incentive would improve response rates for groups with traditionally lower response rates (i.e., non-whites, lower educational attainment, low income and in poor health).

## Methods

### Parent study population

This incentive study was an ancillary study of non-respondents from a larger face-to-face survey of veterans. The study population for the larger main study included all primary care patients at four Veterans Health Administration (VHA) medical centers (Minneapolis, MN; West Los Angeles, CA; Portland, OR; Durham, NC) who were scheduled to have at least one primary care visit during the study recruitment period (June, 2004 through May, 2005) and who did not suffer from a severe cognitive disorder (i.e., Alzheimer's disease, severe dementia, schizophrenia) or blindness, as determined from an initial review of medical records. Invitations to participate in the main study, which included a face-to-face interview at the medical center to assess the patient's health literacy skills and an offer of a $25 cash incentive for completing the interview, were mailed to randomly selected patients at each site and then participants were recruited by phone. Study recruiters telephoned each potential participant to determine their willingness to participate approximately 10 days after the mailed invitations were sent. Six attempts were made to reach participants at different times of day. Patients were classified into willing participants, hard refusers (e.g., did not want anything to do with research), soft refusers (e.g., could not participate because of logistical reasons), and those whom we could not reach by phone or mail. Those who were reached and willing to participate booked a one-hour research appointment, usually on the same day as their scheduled primary care appointment[20]. Institutional Review Boards from the each study site (Minneapolis, MN; West Los Angeles, CA; Portland, OR; Durham, NC) approved the study protocol.

### Ancillary study population

**Figure 1:**
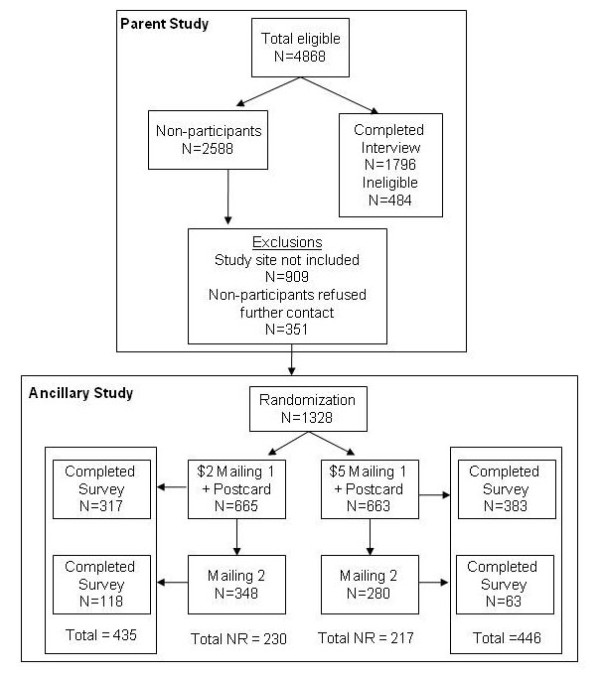
**Participant flow chart for parent and ancillary studies**.

In order to assess the effect of non-participation on prevalence estimates of poor health literacy, eligible patients from the parent study who could not be reached, did not attend their scheduled research appointment, or refused because of transportation, scheduling difficulties, or other conflicts were mailed a one page, plain language, ancillary survey designed to characterize non-responders. This group of parent study non-respondents, as outlined in Figure 1, comprises the sample for this study.

### Data and measurement

Data from the ancillary survey and medical record and administrative sources were used as independent variables in this investigation. Sex, age (< 50, 50-75, > 75), urban/rural residence (determined from U.S. census data), comorbidity history and mental health diagnoses were extracted from VHA administrative and medical record data; therefore, data were available for ancillary survey respondents and non-respondents. Comorbidities were summarized using the Charlson Comorbidity Index score[21] and a measure of mental health diagnoses which categorized individuals into one of three groups: (1) no mental health diagnoses, (2) at least one psychiatric (ICD-9 codes 290-302 and 306-311) or substance abuse related (ICD-9 codes 303-305) diagnosis, or (3) dual diagnosis (psychiatric and substance abuse). Measures of mental health diagnoses were included because they could conceivably affect the accuracy of survey response and are not captured in the Charlson Comorbidity Index. Ancillary survey questions included 4 self-reported health literacy questions[22], marital status (recoded into married versus unmarried), race (white, African American, other), education (≤ high school, some college, ≥ college graduate), employment status (employed versus not employed) and income (recoded into ≥ $20,000, $20-40,000, > $40,000).

### Ancillary survey administration

The ancillary survey was administered by a university-affiliated survey center not associated with the parent study or its investigators. In three of the medical center sites, patients were randomly assigned to either a $2 or $5 prepaid cash incentive condition, and the data from these three sites were used for this paper. The fourth site did not participate in the incentive experiment. Survey packets were mailed using first-class postage to 1,328 patients. Participants were assigned random numbers between 0 and 1 and then based on the number (< 0.5 or > 0.5), participants split into two equal groups. Randomization was performed blindly, with no information about individual cases to affect the distribution. The first survey packet included the cash incentive, a cover letter, a pre-addressed postage-paid business envelope and the 10-item questionnaire. A reminder postcard was mailed approximately one week after the first packet. A second questionnaire packet without an incentive was mailed to any subject who did not return a blank or completed survey within 3-4 weeks of the first mailing. Surveys returned before the mailing of the second questionnaire were categorized "first mailing respondents." Surveys received after this period were categorized "second mailing respondents."

### Analysis

All analysis was completed using SAS version 9.1. To determine the success of the randomization, contrasts of the respondent's demographic and health characteristics by their incentive condition were first compared using chi-square tests. Significant differences found from these results were later controlled for in logistic regressions. Response rates were calculated using American Association of Public Opinion Research (AAPOR) RR1 criteria[23]. Differences in response rates by incentive group were compared by demographic and health factors. Cumulative response rates by incentive group ($2 or $5) were then compared for the "first mailing participants" and then for the "second mailing participants." Logistic regression was then used to calculate the odds of responding to the survey with a $5 versus a $2 incentive, controlling for variables that were not successfully randomized (sex, rural/urban status, and site), as well as incentive. Because other demographic and health characteristics were not significantly different in bivariate analyses, these variables were not included in the regression analyses used to calculate odds ratios.

The cost of each survey packet included materials (1 page survey, outgoing and return envelopes), postage (outgoing and return) and labor. Labor costs per survey were calculated by multiplying the hourly rate, including benefits, in the year the survey was fielded (2005) by the time that employees and supervisors spent stamping, sealing and stuffing packets as well as logging returns and data entry of survey results, divided by the number of surveys sent. Time spent processing returned surveys was calculated for the total response to each mailing, without regard to incentive status. The cost of the reminder postcard includes printing, labor and outgoing postage. The reminder postcard was sent to all recipients of the first mailing and is included in the total cost of that mailing. The cost per completed survey was determined for each round of mailing by dividing the total cost of the mailings by the number of completed surveys returned. Indirect costs were not included.

In order to assess whether ancillary survey respondents were representative of all those to whom the survey was sent, demographic and health status of respondents for each incentive condition and mailing were compared to the entire ancillary study sample.

## Results

### Sample characteristics

Demographic and health characteristics are shown in Table 1. There were no significant differences between the incentive groups in age, race, marital status, Charlson comorbidity score or mental health diagnoses. The $5 incentive group had a higher proportion of men and those with a rural address than the $2 incentive group. Among survey responders, there were no differences in either level of education completed, or self-reported annual income (these data were not available for non-responders).

**Table 1:** Demographic and Health Characteristics by Incentive Condition

Characteristics	$2 incentive %(n)	$5 incentive %(n)	p-value
Age*			
< 50	34.29 (228)	30.32 (201)	0.1924
50-75	33.53 (223)	33.18 (220)	0.8867
> 75	32.18 (214)	36.50 (242)	0.1898
Race*			
Caucasian	68.57 (456)	68.78 (456)	1.000
African American	15.79 (105)	17.65 (117)	0.4206
Other	15.64 (104)	13.57 (90)	0.3148
Sex*			
Female	18.95 (126)	13.88 (92)	0.0213
Male	81.05 (539)	86.12 (571)	0.3368
Education**			
Grade 11 or less	15.96 (64)	21.32 (87)	0.0612
High school grad	30.42 (122)	33.58 (137)	0.8687
Some college/tech deg	33.92 (136)	27.21 (111)	0.1117
College grad	14.21 (57)	13.24 (54)	0.7758
Post grad (MS, PhD)	5.49 (22)	4.66 (19)	0.6394
Income***			
Under $20,000	50.76 (201)	50.00 (192)	0.6498
$20,000-40,000	31.31 (124)	31.25 (244)	0.7979
$40,000-$60,000	10.86 (43)	11.98 (46)	0.7505
Over $60,000	7.07 (28)	6.77 (26)	0.7855
Marital Status*			
Married/Living with Someone	54.44 (362)	54.75 (363)	0.9704
Not Married	45.56 (303)	45.25 (300)	0.9028
Charlson comorbidity score*			
0	46.17 (307)	42.23 (280)	0.2651
1	20.30 (135)	22.47 (284)	0.4061
2+	33.53 (223)	35.29 (234)	0.6069
Mental health diagnoses*			
No mental health diagnosis	46.32 (308)	47.66 (316)	0.7488
Substance abuse only	8.72 (58)	8.75 (58)	1.000
Psychiatric only	28.27 (188)	26.85 (178)	0.6012
Dual diagnosis	16.69 (111)	16.74 (111)	1.000
Rural/Urban Status*			
Urban	94.89 (631)	90.80 (602)	0.4089
Rural	5.11 (34)	9.20 (61)	0.0056

*Data available for full study sample (N for $2 = 665; N for $5 = 663)

**Data only available from ancillary survey respondents(N for $2 = 401; N for $5 = 408)

***Data only available from ancillary survey respondents (N for $2 = 396; N for $5 = 384)

### Response rates

A total of 881 (66%) of the 1,328 patients completed and returned the questionnaire. As shown in Table 2, unadjusted response rates were significantly higher in the $5 incentive group than the $2 incentive group after the first mailing and reminder postcard (58% and 48%, p = 0.0002). (Table 2). There were no significant differences in response rates after the second survey mailing. The final response rates for the $5 and $2 incentives were 67% and 65%, respectively (p = 0.47). While response rates differed significantly across all demographic and health categories after the first mailing, with older, white, married and male participants, and those with two or more physical comorbidities and no mental health conditions having higher response rates, cumulatively, after both mailings, response rates showed no significant differences across demographic categories.

**Table 2:** Unadjusted and Adjusted odds of response by patient characteristics and incentive condition

	First Mailing/Reminder postcard				Final Response			
	$2	$5	Unadjusted p-value*	Adjusted odds of responding with $5 incentive vs. $2 incentive **	$2	$5	p-value*	Adjusted odds of responding with $5 incentive vs. $2 incentive**
Overall Response Rate (Cumulative)	47.67% N = 317	57.77% N = 383	0.0002	**1.50 (1.21-1.86)**	65.41% N = 435	67.27% N = 446	0.4741	1.09 (0.87-1.36)
**Age**			0.0007				0.7525	
< 50	32.02%	41.29%		**1.49 (1.01-2.22)**	49.56%	50.25%		1.03 (0.70-1.50)
50-75	54.71%	62.73%		1.39 (0.95-2.04)	71.30%	72.73%		1.07 (0.71-1.63)
> 75	57.01%	66.94%		**1.53 (1.04-2.23)**	76.17%	76.45%		1.02 (0.66-1.57)
**Race**			0.0002				0.6142	
Caucasian	58.33%	68.42%		**1.55 (1.18-2.03)**	78.07%	78.07%		1.00 (0.73-1.37)
African American	30.48%	44.44%		**1.83 (1.05-3.17)**	52.38%	56.41%		1.77 (0.69-2.00)
Other	18.27%	21.11%		1.20 (0.59-2.43)	23.08%	26.67%		1.21 (0.63-2.33)
**Sex**			0.0008				0.6642	
Female	31.75%	41.30%		1.51 (0.86-2.65)	56.35%	51.09%		.081 (0.47-1.39)
Male	51.39%	60.42%		**1.44 (1.14-1.83)**	67.53%	69.88%		1.12 (0.87-1.44)
**Marital Status**			0.0002				0.4799	
Married/Living with someone	53.04%	69.70%		**2.04 (1.50-2.76)**	72.38%	76.58%		1.25 (0.89-1.74)
Not Married	41.25%	43.33%		1.09 (0.79-1.50)	57.10%	56.00%		0.96 (0.69-1.32)
**Charlson Comorbidity Score**			0.0003				0.5408	
0	43.97%	52.86%		**1.43 (1.03-1.98)**	60.26%	63.21%		1.13 (0.81-1.58)
1	44.44%	29.23%		1.57 (0.98-2.51)	64.44%	34.86%		1.09 (0.67-1.78)
2+	54.71%	64.96%		**1.53 (1.05-2.24)**	73.09%	72.65%		0.98 (0.65-1.48)
**Mental health diagnoses**			0.0003				0.4929	
No Mental Health Diagnosis	54.55%	62.66%		**1.40 (1.02-1.92)**	70.78%	72.47%		1.09 (0.77-1.54)
Substance abuse only	41.38%	58.62%		2.01 (0.96-4.20)	60.34%	68.97%		1.46 (0.68-3.14)
Psychiatric only	43.09%	55.62%		**1.66 (1.10-2.50)**	64.89%	64.04%		0.96 (0.63-1.48)
Dual diagnosis	39.64%	46.85%		1.34 (0.79-2.29)	54.05%	56.76%		1.12 (0.66-1.89)
**Rural/Urban Status**			0.0007				0.6519	
Urban	46.43%	55.98%		**1.47 (1.17-1.84)**	64.82%	65.45%		1.03 (0.81-1.30)
Rural	70.59%	75.41%		1.28 (0.50-3.27)	76.47%	85.25%		1.78 (0.61-5.14)

*p-values from chi-square test.

**Logistic regression model included predictor, sex, rural/urban status; bolded odds ratios are statistically significant.

These same patterns persisted after adjusting for urban/rural status and sex. After the first mailing, adjusted odds of recipients of the $5 incentive responding versus recipients of the $2 incentive responding show that, overall, recipients of the $5 incentive were 50% more likely to respond than those who received the $2 incentive (Table 2). Odds of the $5 recipients responding were significantly higher within most sub-groups, except for women, those aged 50-75, not married, those with few physical comorbidities, substance use or substance use/psychiatric diagnoses, and those living in a rural area. After the final mailing, however, there were no significant differences in odds of responding across any of the demographic categories.

Table 3 shows the results of the cost analysis. The total cost per survey for the first mailing, which included the survey packet, the $2 or $5 incentive and the reminder postcard was $5.25 for the $2 incentive group and $8.25 for the $5 group; the cost of the second mailing was $2.40 per survey. The cost per completed survey (cost of the mailings\number of surveys returned) overall was lower in the $2 incentive group than the $5 group after both the first and second mailing.

**Table 3:** Total costs comparing $2 and $5 conditions and one versus two mailings

	$2 condition	$5 condition
First Mailing-total cost per participant*	$5.25	$8.25
First mailing response rate	47.67%	57.77%
Total cost/returned survey ($)	$11.01	$14.28
Second Mailing-total cost per participant**	$2.40	$2.40
Second mailing response rate	33.91%	22.50%
Total cost/returned survey ($)	$7.08	$10.67
**Final Response**		
Cumulative response rate	65.41%	67.27%
Total cost/returned survey ($)	$9.95	$13.77

*total cost per survey included survey packet, incentive and reminder postcard

**total cost per survey included survey packet only

Table 4 displays the representativeness of respondents to the total ancillary study population. These data show that the two incentive conditions provide equally representative samples with the exception of urban/rural status, where the $5 incentive condition, but not the $2 condition, produced a respondent population more heavily weighted toward those living in rural areas than the population sampled.

**Table 4:** Representativeness of Responders on Demographic Characteristics

		$2 incentive			$5 Incentive		
Characteristics	Ancillary study population	$2 incentive population	1st Mailing	**2**^**nd **^**Mailing**	$5 incentive population	**1**^**st **^**Mailing**	**2**^**nd **^**Mailing**
	(N = 1328)	(N = 665)	(N = 317)	(N = 435)	(N = 663)	(N = 383)	(N = 446)
**Age**			**xx**	**xx**		**xx**	**xx**
< 50	32.30 (429)	34.29 (228)	23.03 (73)	25.98 (113)	30.32 (201)	21.67 (83)	22.65 (101)
50-75	33.36 (443)	33.53 (223)	38.49 (122)	36.55 (159)	33.18 (220)	36.03 (138)	35.87 (160)
> 75	34.34 (456)	32.18 (214)	38.49 (122)	37.47 (163)	36.50 (242)	42.30 (162)	41.48 (185)
**Race**			**xx**	**xx**		**xx**	**xx**
Caucasian	68.67 (912)	68.57 (456)	83.91 (266)	81.84 (356)	68.78 (456)	81.46 (312)	79.82 (356)
African American	16.72 (222)	15.79 (105)	10.09 (32)	12.64 (55)	17.65 (117)	13.58 (52)	14.80 (66)
Other	14.61 (194)	15.64 (104)	5.99 (19)	5.52 (24)	13.57 (90)	4.96 (19)	5.38 (24)
**Sex**			**x**			**xx**	**xx**
Female	16.42 (218)	18.95 (126)	12.62 (40)	16.32 (71)	13.88 (92)	9.92 (38)	10.54 (47)
Male	83.58 (1110)	81.05 (539)	87.38 (277)	83.68 (364)	86.12 (571)	90.08 (345)	89.46 (399)
**Marital Status**			**x**	**xx**		**xx**	**xx**
Married/Living with Someone	54.59 (725)	54.44 (362)	60.57 (192)	60.23 (262)	54.75 (363)	66.06 (253)	62.33 (278)
Not Married	45.41 (603)	45.56 (303)	39.43 (125)	39.77 (173)	45.25 (300)	33.94 (130)	37.67 (168)
**Charlson comorbidity score**							
0	44.20 (587)	46.17 (307)	42.59 (135)	42.53 (185)	42.23 (280)	38.64 (148)	39.69 (177)
1	21.39 (284)	20.30 (135)	18.93 (60)	20.00 (87)	22.47 (284)	21.67 (83)	22.20 (99)
2+	34.41 (457)	33.53 (223)	38.49 (122)	37.47 (163)	35.29 (234)	39.69 (152)	38.12 (170)
**Mental health diagnoses**							
No mental health diagnosis	46.99 (624)	46.32 (308)	53.00 (168)	50.11 (218)	47.66 (316)	51.70 (198)	51.35 (229)
Substance abuse only	8.73 (116)	8.72 (58)	7.57 (24)	8.05 (35)	8.75 (58)	8.88 (34)	8.97 (40)
Psychiatric only	27.56 (366)	28.27 (188)	25.55 (81)	28.05 (122)	26.85 (178)	25.85 (99)	25.56 (114)
Dual diagnosis	16.72 (222)	16.69 (111)	13.88 (44)	13.79 (60)	16.74 (111)	13.58 (52)	14.13 (63)
**Rural/Urban Status**						**xx**	**xx**
Urban	92.85 (1233)	94.89 (631)	92.43 (293)	94.02 (409)	90.80 (602)	87.99 (337)	88.34 (394)
Rural	7.15 (95)	5.11 (34)	7.57 (24)	5.98 (26)	9.20 (61)	12.01 (46)	11.66 (52)

x p < 0.10 from overall non-responder population

xx p < 0.05 from overall non-responder population

## Discussion

Reasons for not participating in research are numerous, but typically are demarcated by researchers not being able to reach participants (e.g., incorrect or unavailable address or phone number) or participants not interested in (e.g., refusals) or not able to complete (e.g., poor literacy, limited capacity to understand study protocol) a study[24,25]. Gathering as much information as possible about non-responders in order to assess potential bias of study results is often endorsed by survey methodologists[26]. One option is to re-contact non-responders and ask to gather a small set of critical data that will allow for a basic description of the non-responders. Re-contacting initial non-responders, however, is potentially costly, especially because it is unclear what the likelihood is that non-responders will convert to responders; therefore, maximizing the effectiveness of incentives for the greatest response is important. In this study we evaluate if monetary incentives and multiple mailings are effective methods for increasing response rates in a sample of participants that had been difficult to reach or unavailable or unwilling to participate in a longer, more complex study that required face-to-face interviews.

Survey methodologists recommend the use of incentives and multiple mailings to increase response rates[8,27,28] and our findings support this approach. Like previous studies of patient populations, our results show that a $5 incentive produces higher overall response rates than a $2 incentive[19,21]. However, our study also showed that, while the response rate in the $5 incentive group was significantly higher after the first mailing, the response rates were relatively equal after a second mailing. We also found that the respondents across incentive conditions, with the exception of urban/rural status, represented the overall ancillary study population. Parkes et al, in a case-control study, randomized 2561 controls to receive no incentive, $2 or $5 and had comparable findings to ours: those receiving a $5 incentive, even after multiple mailing and reminder phone calls, had comparable response rates to those receiving a $2 incentive, although the non-significant difference in response rates (4.2%) was slightly higher than we observed[21]. Shaw et al., in their community-based survey of 1800 health plan enrollees, tested differences in survey response rates with either $2 or $5 cash incentives and found that although a $5 incentive yielded a higher response rate after one mailing, with multiple mailings the response rate from the $2 incentive was reasonable and adequate[19]. The consistency of our findings with others suggests that regardless of whether participants are being contacted for the first time, as was the case in Parkes' and Shaw's studies, or have been difficult to reach previously or hesitant to participate in prior research, as was the case in our study, the conclusions are relatively similar: with a restricted timeline, a $5 incentive will provide a higher response rates for most groups, yet if time permits, a $2 incentive with multiple contacts may be sufficient to yield acceptable response rates.

Our cost analysis suggests that it may be more cost effective to have multiple contacts than to provide an increased incentive in order to maximize response rates. We found the best "bang for the buck" was a smaller incentive with two mailings when costs were compared relative to response rates. With an initial higher response rate for the $5 group after the first mailing, one might conclude that the extra incentive was well spent. However, the total cost per returned survey shows that response rate was not high enough to decrease the overall costs. Moreover, the cost of the second survey packet ($2.40) was less than the $3 difference in incentives, making the $2 incentive with multiple mailings the more economical method. What we are not able to distinguish from our analysis, however, is why this is the case. While it is likely that some proportion of respondents will respond quickly regardless of the incentive amount, it is not clear if the $5 incentive also entices some of those who otherwise would hesitate to respond more promptly. If the probability of response does increase within this hesitant group, then we would expect the respondents to the first mailing in the $2 incentive condition to include mostly those who would respond regardless of the incentive amount. Therefore, the effect of a second mailing may be stronger than the effect of increasing the incentive by $3 in the first mailing for the $2 incentive group because the remaining participants include more hesitant non-responders.

While several studies have shown that larger incentives significantly improve the odds of responding for groups with low-income, low educational attainment, gender and non-white race[8,29], fewer have investigated differences in other sub-groups, such as marital status or poor physical or mental health, factors that may, in addition to the incentive, affect either external support or personal capacity for responding to a survey. Like the cumulative response rates, our findings show higher odds of responding with a $5 incentive within every demographic and health sub-group after the first mailing, but no significant differences overall after both mailings, suggesting that with greater response time and multiple mailings, the effect of an increased incentive is negligible. It should be noted, however, that although there are no significant differences across incentive groups after both mailings, the absolute response rate for those less than 50 years, women, non-whites, those unmarried or with chronic physical or mental illness is low. Our data suggest that, perhaps with the exception of those with chronic physical or mental illness, these groups are underrepresented compared to the overall population and it is possible that neither incentives nor multiple mailings may entice them to participate. These results suggest that additional studies need to be designed in order understand reasons for high non-participation rates in hard-to-reach groups and that more innovative strategies, including but not limited to incentives, need to be developed and tested to encourage adequate representation of these populations in research.

This study is tempered by a number of limitations. First, with the design of our study we assume that response is based on incentive condition, but other unmeasured factors, could have varied by condition and accounted for some of the differences in response rates or timing of response. Second, it is unclear why randomization was not successful, with more women and fewer rural residents in the $2 incentive group. Because randomization does not guarantee balance in any variable among treatment groups, there is always some amount of chance that imbalance between groups will occur. In order to account for this limitation in our randomization strategy, we adjusted for these variables in our analyses. Third, while we have some demographic and health data on ancillary survey non-respondents, we do not have income or education for the full sample, limiting our ability to assess the effect of these variables on non-response bias.

## Conclusions

We conclude that for studies with a limited budget, increasing the incentive from $2 to $5 is less effective than a subsequent mailing. If time is limited or only one mailing is possible, a $5 incentive leads to higher initial response. However, regardless of incentives and number of mailings, some demographic groups may not respond and innovative strategies are needed assure adequate representation of these groups.

## Competing interests

The authors declare that they have no competing interests.

## Authors' contributions

All authors have read and approved the final manuscript. JMG, MRP, SN, ABS, EH, JS and JPG developed the study concept and design. JMG and JS acquired the data. JMG, MRP, SN, ABS, EH, JS, and JPG analyzed and interpreted the data. JMG, MRP, ABS, EH and JS drafted the manuscript and all authors provided critical revisions for important intellectual content. The study was supervised by JMG.

## Pre-publication history

The pre-publication history for this paper can be accessed here:

http://www.biomedcentral.com/1471-2288/11/81/prepub
